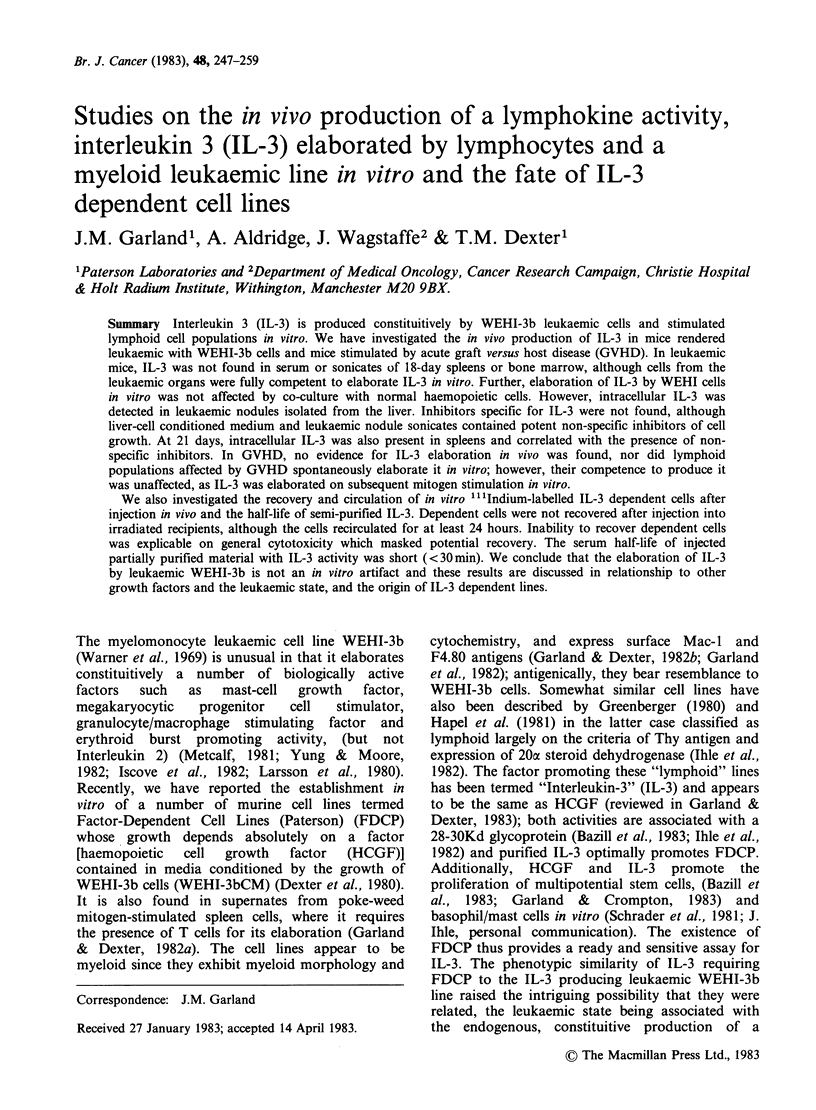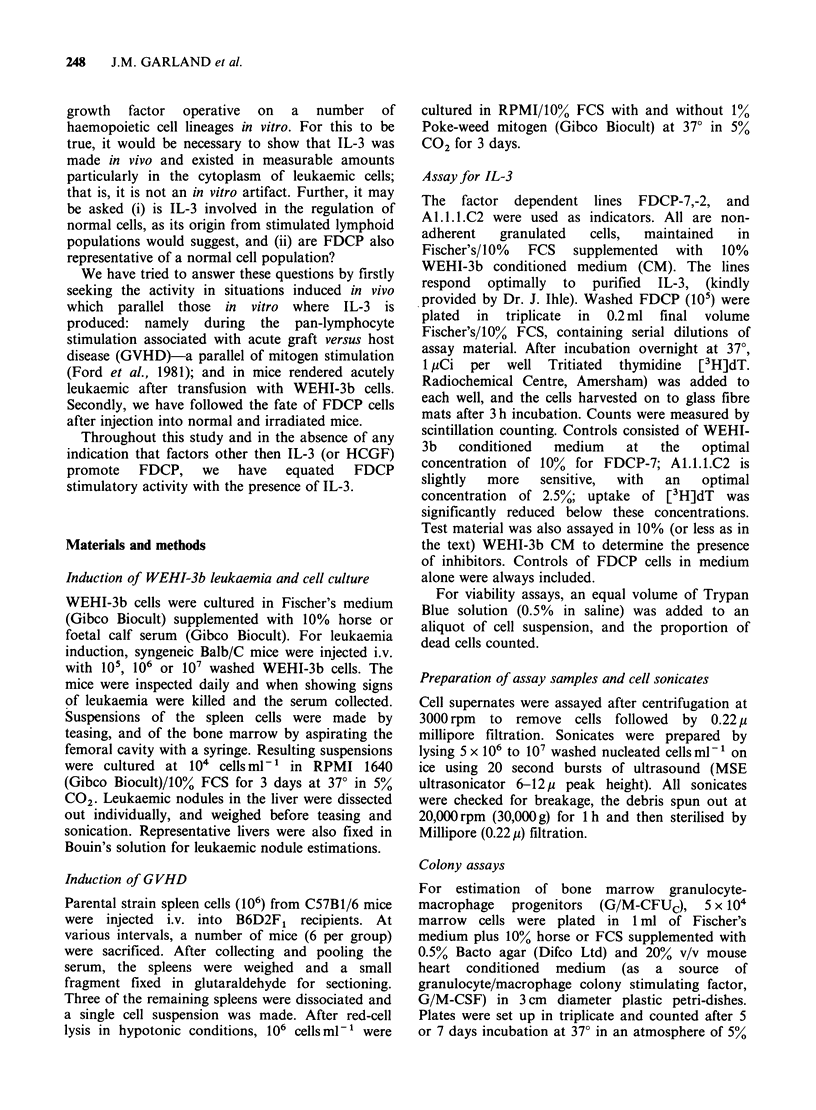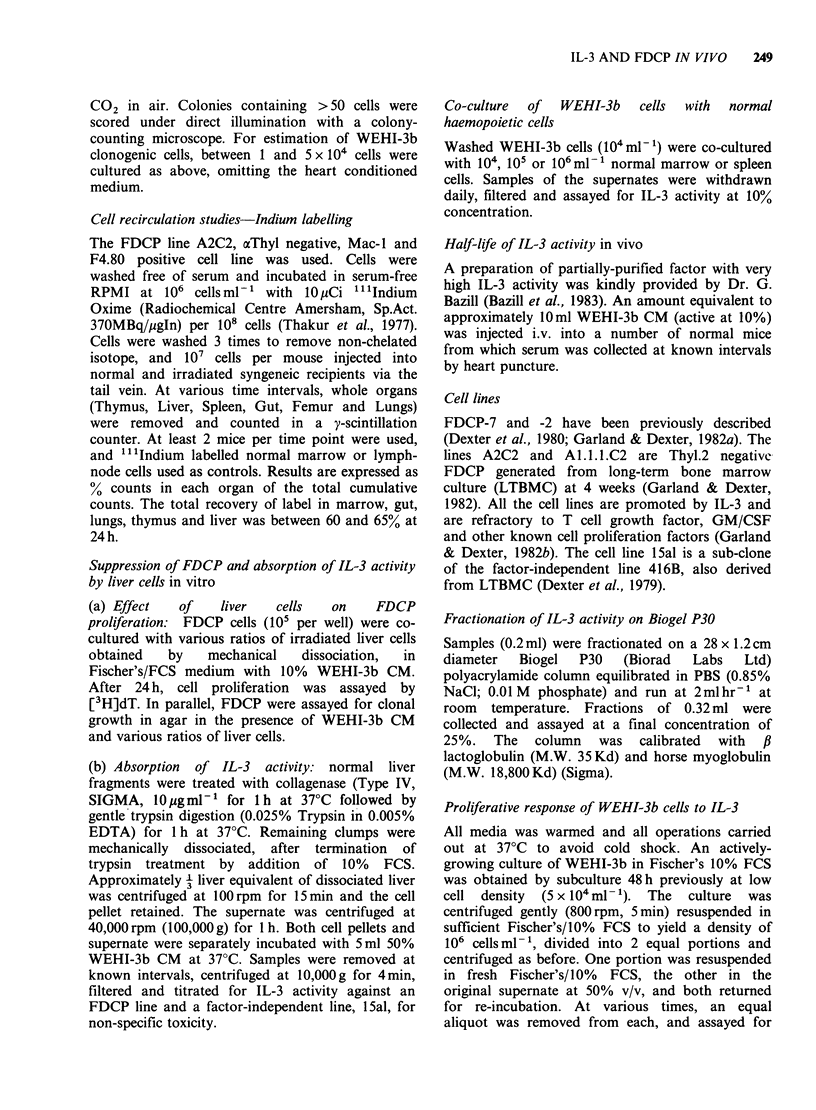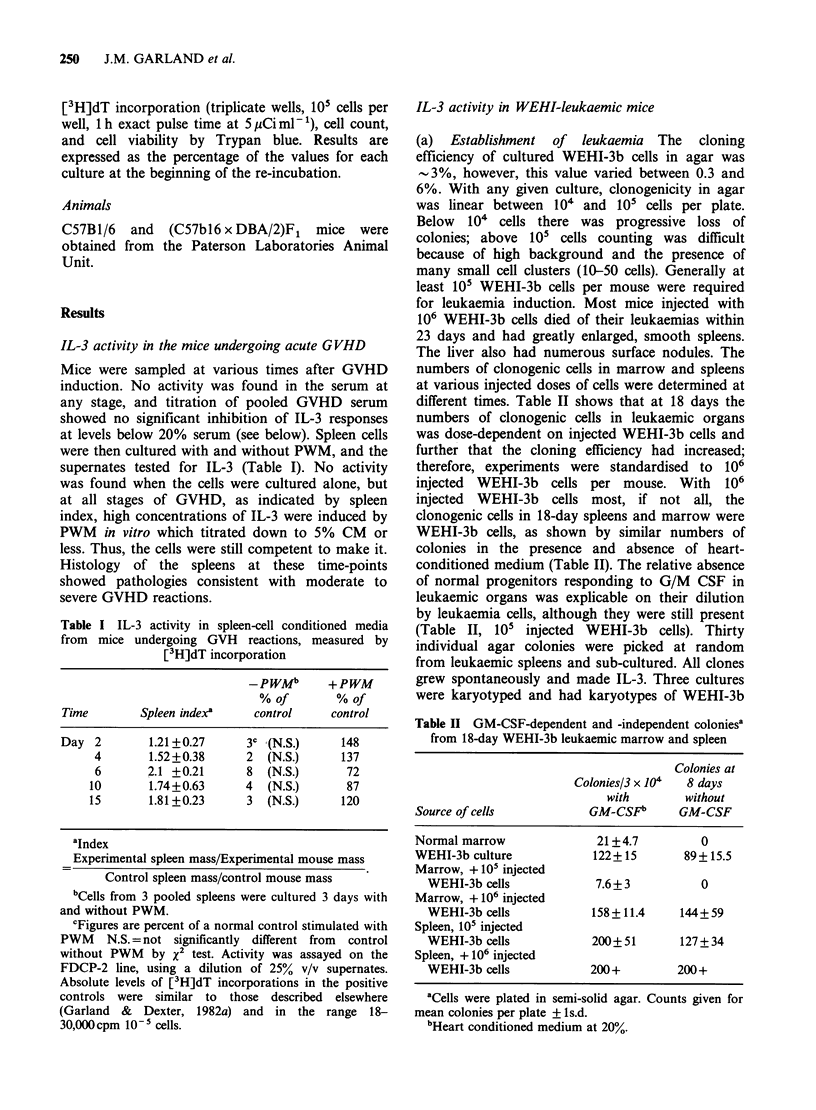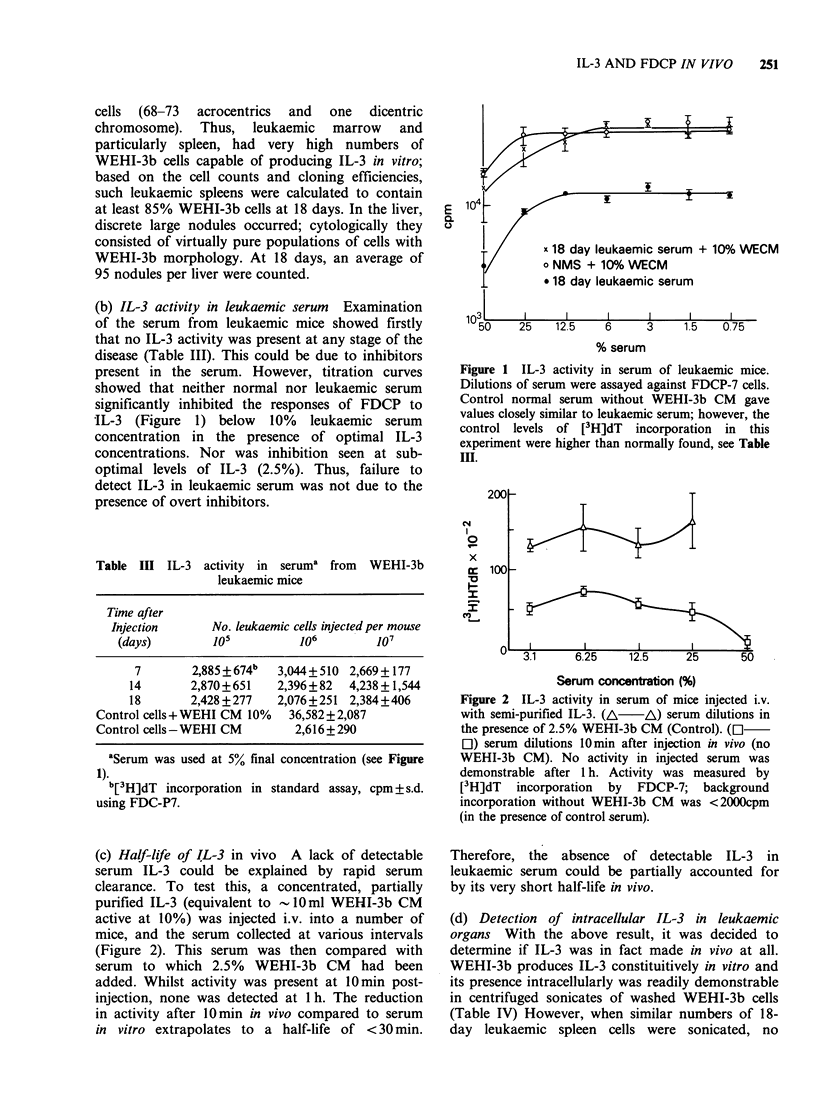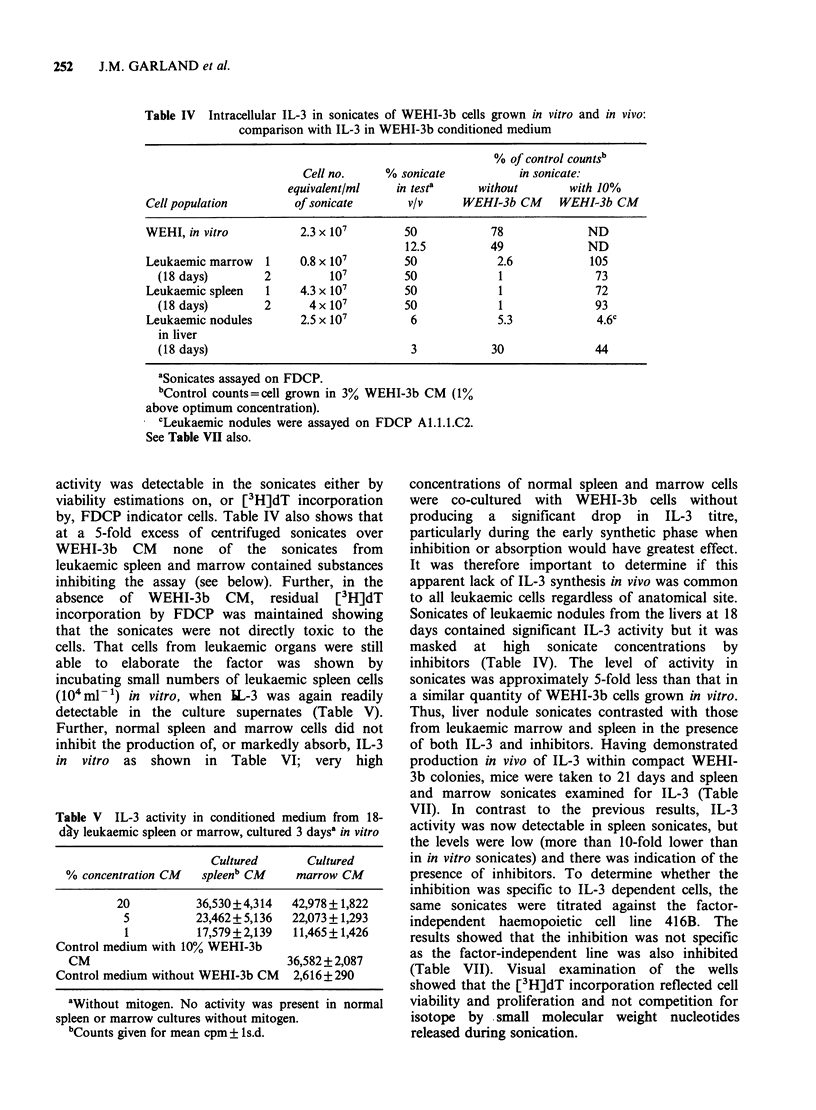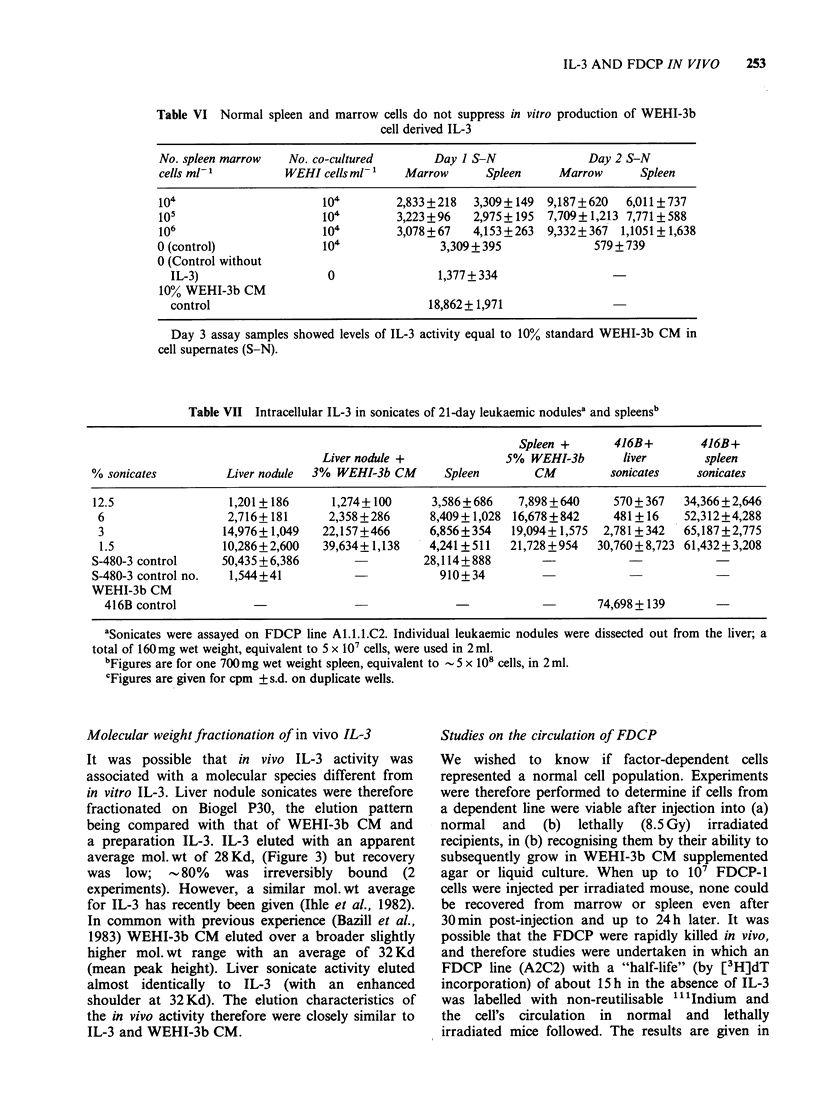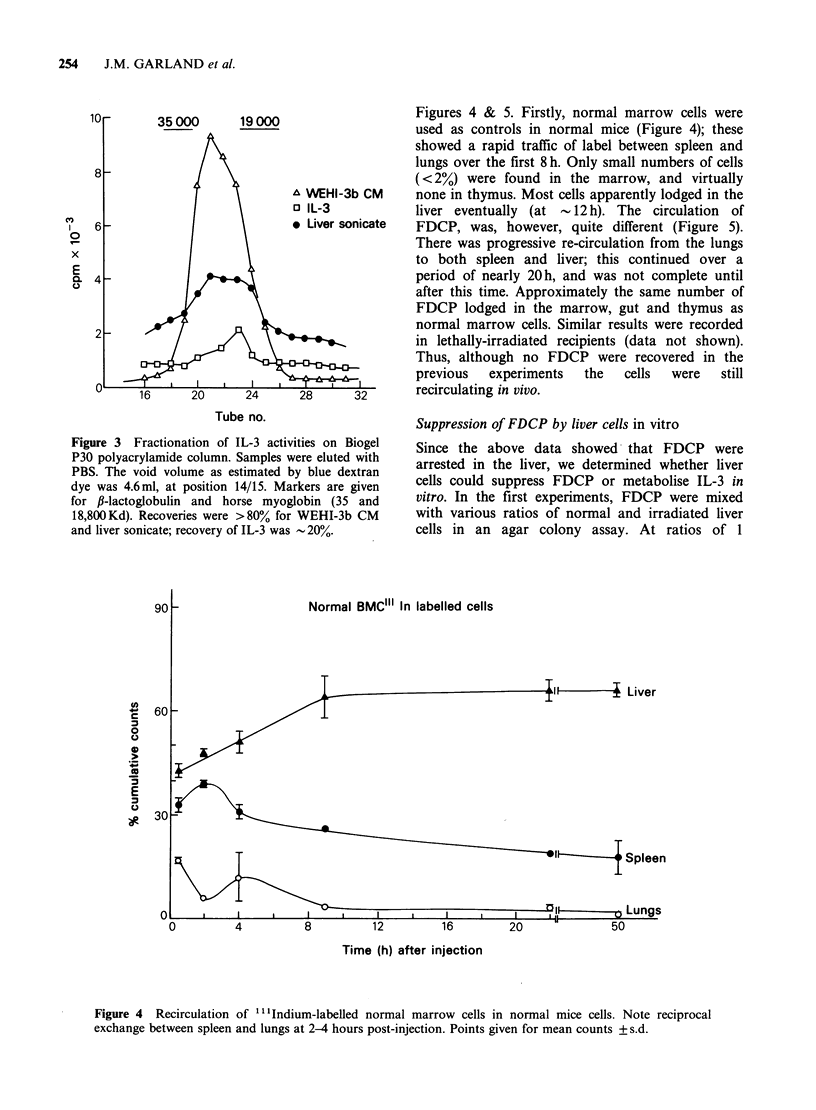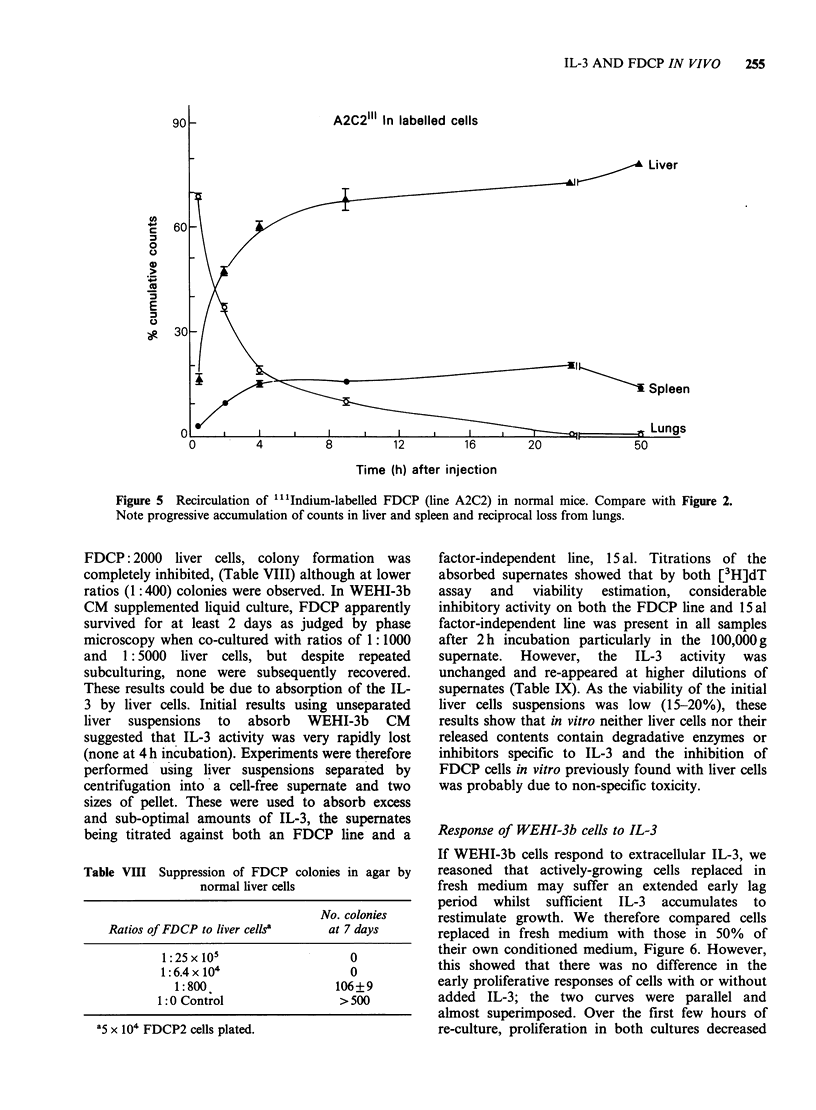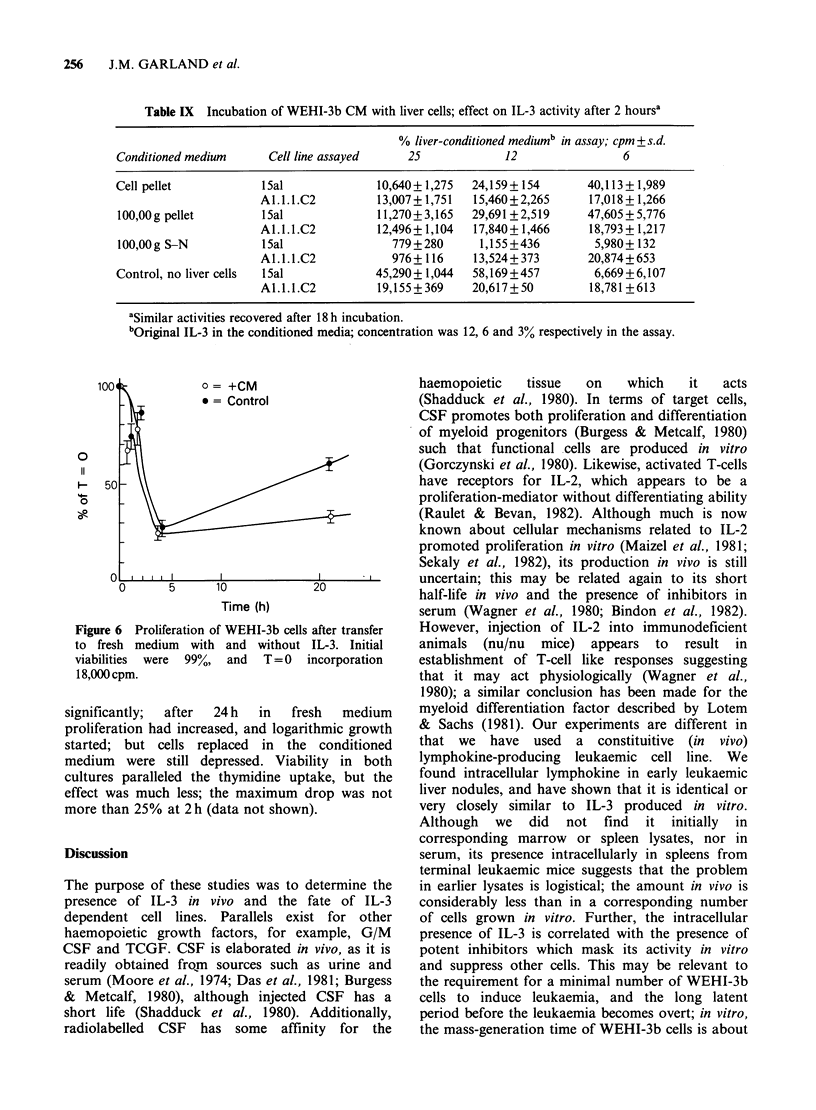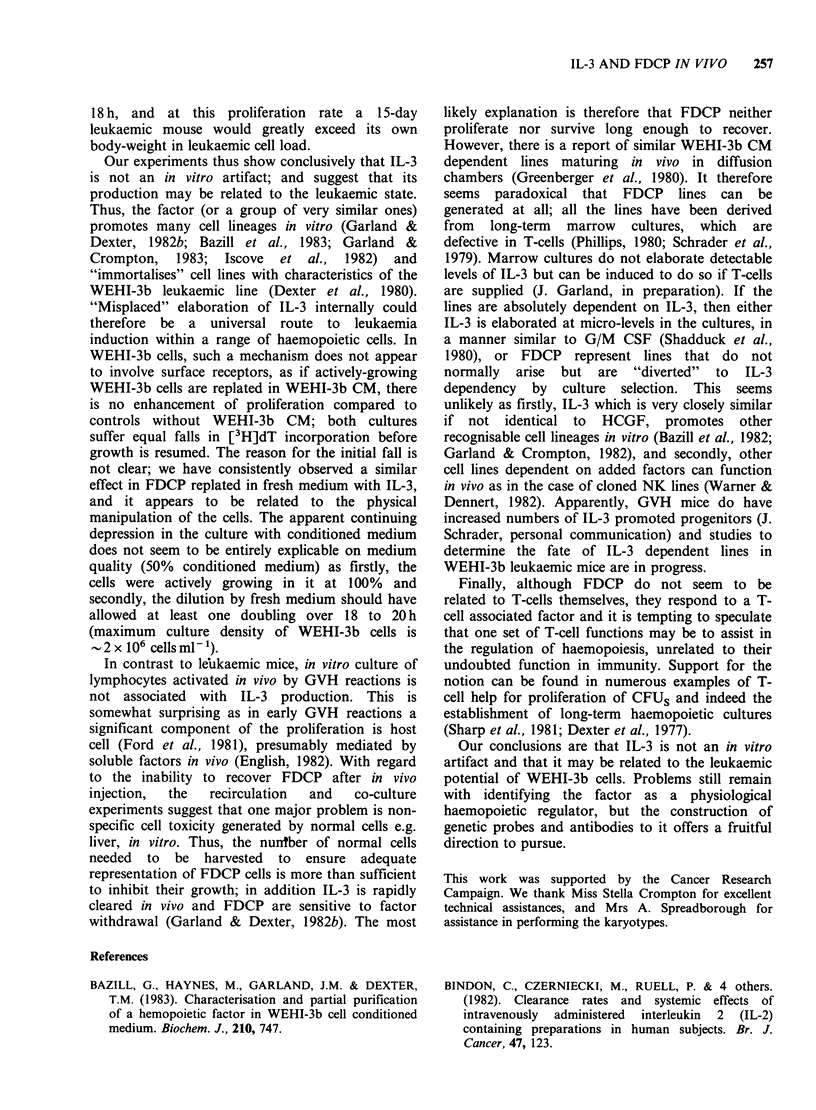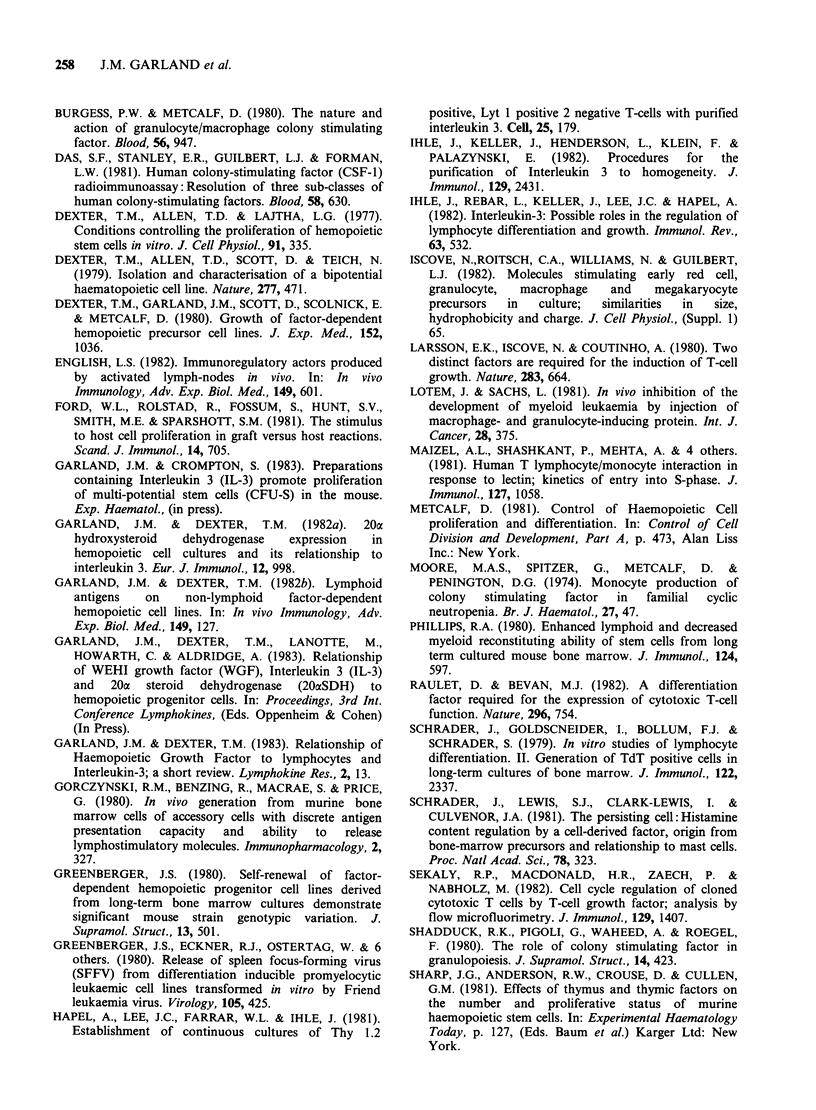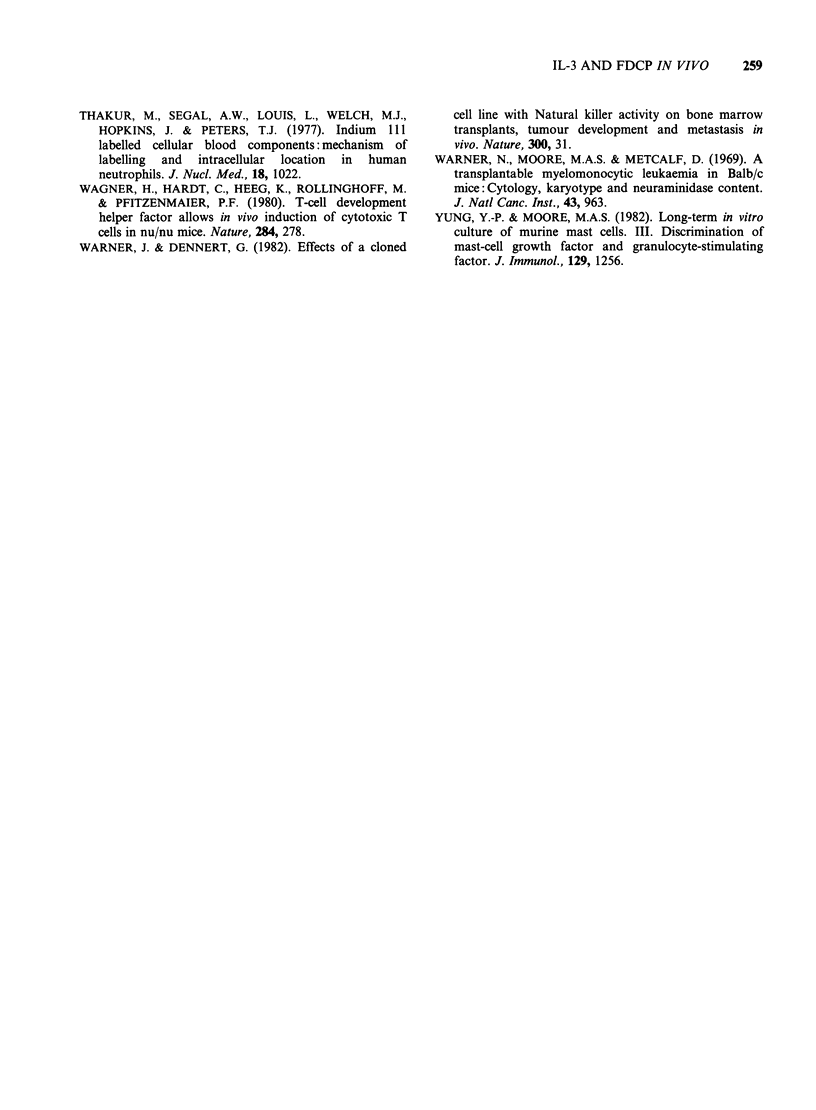# Studies on the in vivo production of a lymphokine activity, interleukin 3 (IL-3) elaborated by lymphocytes and a myeloid leukaemic line in vitro and the fate of IL-3 dependent cell lines.

**DOI:** 10.1038/bjc.1983.180

**Published:** 1983-08

**Authors:** J. M. Garland, A. Aldridge, J. Wagstaffe, T. M. Dexter

## Abstract

Interleukin 3 (IL-3) is produced constitutively by WEHI-3b leukaemic cells and stimulated lymphoid cell populations in vitro. We have investigated the in vivo production of IL-3 in mice rendered leukaemic with WEHI-3b cells and mice stimulated by acute graft versus host disease (GVHD). In leukaemic mice, IL-3 was not found in serum or sonicates of 18-day spleens or bone marrow, although cells from the leukaemic organs were fully competent to elaborate IL-3 in vitro. Further, elaboration of IL-3 by WEHI cells in vitro was not affected by co-culture with normal haemopoietic cells. However, intracellular IL-3 was detected in leukaemic nodules isolated from the liver. Inhibitors specific for IL-3 were not found, although liver-cell conditioned medium and leukaemic nodule sonicates contained potent non-specific inhibitors of cell growth. At 21 days, intracellular IL-3 was also present in spleens and correlated with the presence of non-specific inhibitors. In GVHD, no evidence for IL-3 elaboration in vivo was found, nor did lymphoid populations affected by GVHD spontaneously elaborate it in vitro; however, their competence to produce it was unaffected, as IL-3 was elaborated on subsequent mitogen stimulation in vitro. We also investigated the recovery and circulation of in vitro 111Indium-labelled IL-3 dependent cells after injection in vivo and the half-life of semi-purified IL-3. Dependent cells were not recovered after injection into irradiated recipients, although the cells recirculated for at least 24 hours. Inability to recover dependent cells was explicable on general cytotoxicity which masked potential recovery. The serum half-life of injected partially purified material with IL-3 activity was short (less than 30 min). We conclude that the elaboration of IL-3 by leukaemic WEHI-3b is not an in vitro artifact and these results are discussed in relationship to other growth factors and the leukaemic state, and the origin of IL-3 dependent lines.


Br. J. Cancer (1983), 48, 247–259

### Studies on the *in vivo* production of a lymphokine activity, interleukin 3 (IL-3) elaborated by lymphocytes and a myeloid leukaemic line *in vitro* and the fate of IL-3 dependent cell lines

J.M. Garland[1], A. Aldridge, J. Wagstaffe[2] & T.M. Dexter[1]

[1]*Paterson Laboratories and* [2]*Department of Medical Oncology, Cancer Research Campaign, Christie Hospital & Holt Radium Institute, Withington, Manchester M20 9BX.*

**Summary** Interleukin 3 (IL-3) is produced constituitively by WEHI-3b leukaemic cells and stimulated lymphoid cell populations *in vitro*. We have investigated the *in vivo* production of IL-3 in mice rendered leukaemic with WEHI-3b cells and mice stimulated by acute graft *versus* host disease (GVHD). In leukaemic mice, IL-3 was not found in serum or sonicates of 18-day spleens or bone marrow, although cells from the leukaemic organs were fully competent to elaborate IL-3 *in vitro*. Further, elaboration of IL-3 by WEHI cells *in vitro* was not affected by co-culture with normal haemopoietic cells. However, intracellular IL-3 was detected in leukaemic nodules isolated from the liver. Inhibitors specific for IL-3 were not found, although liver-cell conditioned medium and leukaemic nodule sonicates contained potent non-specific inhibitors of cell growth. At 21 days, intracellular IL-3 was also present in spleens and correlated with the presence of non-specific inhibitors. In GVHD, no evidence for IL-3 elaboration *in vivo* was found, nor did lymphoid populations affected by GVHD spontaneously elaborate it *in vitro*; however, their competence to produce it was unaffected, as IL-3 was elaborated on subsequent mitogen stimulation *in vitro*.

We also investigated the recovery and circulation of *in vitro* $^{111}$Indium-labelled IL-3 dependent cells after injection *in vivo* and the half-life of semi-purified IL-3. Dependent cells were not recovered after injection into irradiated recipients, although the cells recirculated for at least 24 hours. Inability to recover dependent cells was explicable on general cytotoxicity which masked potential recovery. The serum half-life of injected partially purified material with IL-3 activity was short ($<30$ min). We conclude that the elaboration of IL-3 by leukaemic WEHI-3b is not an *in vitro* artifact and these results are discussed in relationship to other growth factors and the leukaemic state, and the origin of IL-3 dependent lines.

The myelomonocyte leukaemic cell line WEHI-3b (Warner *et al.*, 1969) is unusual in that it elaborates constituitively a number of biologically active factors such as mast-cell growth factor, megakaryocytic progenitor cell stimulator, granulocyte/macrophage stimulating factor and erythroid burst promoting activity, (but not Interleukin 2) (Metcalf, 1981; Yung & Moore, 1982; Iscove *et al.*, 1982; Larsson *et al.*, 1980). Recently, we have reported the establishment *in vitro* of a number of murine cell lines termed Factor-Dependent Cell Lines (Paterson) (FDCP) whose growth depends absolutely on a factor [haemopoietic cell growth factor (HCGF)] contained in media conditioned by the growth of WEHI-3b cells (WEHI-3bCM) (Dexter *et al.*, 1980). It is also found in supernates from poke-weed mitogen-stimulated spleen cells, where it requires the presence of T cells for its elaboration (Garland & Dexter, 1982*a*). The cell lines appear to be myeloid since they exhibit myeloid morphology and cytochemistry, and express surface Mac-1 and F4.80 antigens (Garland & Dexter, 1982*b*; Garland *et al.*, 1982); antigenically, they bear resemblance to WEHI-3b cells. Somewhat similar cell lines have also been described by Greenberger (1980) and Hapel *et al.* (1981) in the latter case classified as lymphoid largely on the criteria of Thy antigen and expression of 20$\alpha$ steroid dehydrogenase (Ihle *et al.*, 1982). The factor promoting these "lymphoid" lines has been termed "Interleukin-3" (IL-3) and appears to be the same as HCGF (reviewed in Garland & Dexter, 1983); both activities are associated with a 28-30Kd glycoprotein (Bazill *et al.*, 1983; Ihle *et al.*, 1982) and purified IL-3 optimally promotes FDCP. Additionally, HCGF and IL-3 promote the proliferation of multipotential stem cells, (Bazill *et al.*, 1983; Garland & Crompton, 1983) and basophil/mast cells *in vitro* (Schrader *et al.*, 1981; J. Ihle, personal communication). The existence of FDCP thus provides a ready and sensitive assay for IL-3. The phenotypic similarity of IL-3 requiring FDCP to the IL-3 producing leukaemic WEHI-3b line raised the intriguing possibility that they were related, the leukaemic state being associated with the endogenous, constituitive production of a

Correspondence: J.M. Garland

Received 27 January 1983; accepted 14 April 1983.

© The Macmillan Press Ltd., 1983

growth factor operative on a number of haemopoietic cell lineages *in vitro*. For this to be true, it would be necessary to show that IL-3 was made *in vivo* and existed in measurable amounts particularly in the cytoplasm of leukaemic cells; that is, it is not an *in vitro* artifact. Further, it may be asked (i) is IL-3 involved in the regulation of normal cells, as its origin from stimulated lymphoid populations would suggest, and (ii) are FDCP also representative of a normal cell population?

We have tried to answer these questions by firstly seeking the activity in situations induced *in vivo* which parallel those *in vitro* where IL-3 is produced: namely during the pan-lymphocyte stimulation associated with acute graft *versus* host disease (GVHD)—a parallel of mitogen stimulation (Ford *et al.*, 1981); and in mice rendered acutely leukaemic after transfusion with WEHI-3b cells. Secondly, we have followed the fate of FDCP cells after injection into normal and irradiated mice.

Throughout this study and in the absence of any indication that factors other then IL-3 (or HCGF) promote FDCP, we have equated FDCP stimulatory activity with the presence of IL-3.

### Materials and methods

### *Induction of WEHI-3b leukaemia and cell culture*

WEHI-3b cells were cultured in Fischer's medium (Gibco Biocult) supplemented with 10% horse or foetal calf serum (Gibco Biocult). For leukaemia induction, syngeneic Balb/C mice were injected i.v. with $10^5$, $10^6$ or $10^7$ washed WEHI-3b cells. The mice were inspected daily and when showing signs of leukaemia were killed and the serum collected. Suspensions of the spleen cells were made by teasing, and of the bone marrow by aspirating the femoral cavity with a syringe. Resulting suspensions were cultured at $10^4$ cells $ml^{-1}$ in RPMI 1640 (Gibco Biocult)/10% FCS for 3 days at 37° in 5% $CO_2$. Leukaemic nodules in the liver were dissected out individually, and weighed before teasing and sonication. Representative livers were also fixed in Bouin's solution for leukaemic nodule estimations.

### *Induction of GVHD*

Parental strain spleen cells ($10^6$) from C57B1/6 mice were injected i.v. into $B6D2F_1$ recipients. At various intervals, a number of mice (6 per group) were sacrificed. After collecting and pooling the serum, the spleens were weighed and a small fragment fixed in glutaraldehyde for sectioning. Three of the remaining spleens were dissociated and a single cell suspension was made. After red-cell lysis in hypotonic conditions, $10^6$ cells $ml^{-1}$ were cultured in RPMI/10% FCS with and without 1% Poke-weed mitogen (Gibco Biocult) at 37° in 5% $CO_2$ for 3 days.

### *Assay for IL-3*

The factor dependent lines FDCP-7,-2, and A1.1.1.C2 were used as indicators. All are non-adherent granulated cells, maintained in Fischer's/10% FCS supplemented with 10% WEHI-3b conditioned medium (CM). The lines respond optimally to purified IL-3, (kindly provided by Dr. J. Ihle). Washed FDCP ($10^5$) were plated in triplicate in 0.2 ml final volume Fischer's/10% FCS, containing serial dilutions of assay material. After incubation overnight at 37°, 1 $\mu$Ci per well Tritiated thymidine [$^3$H]dT. Radiochemical Centre, Amersham) was added to each well, and the cells harvested on to glass fibre mats after 3 h incubation. Counts were measured by scintillation counting. Controls consisted of WEHI-3b conditioned medium at the optimal concentration of 10% for FDCP-7; A1.1.1.C2 is slightly more sensitive, with an optimal concentration of 2.5%; uptake of [$^3$H]dT was significantly reduced below these concentrations. Test material was also assayed in 10% (or less as in the text) WEHI-3b CM to determine the presence of inhibitors. Controls of FDCP cells in medium alone were always included.

For viability assays, an equal volume of Trypan Blue solution (0.5% in saline) was added to an aliquot of cell suspension, and the proportion of dead cells counted.

### *Preparation of assay samples and cell sonicates*

Cell supernates were assayed after centrifugation at 3000 rpm to remove cells followed by 0.22 $\mu$ millipore filtration. Sonicates were prepared by lysing $5 \times 10^6$ to $10^7$ washed nucleated cells $ml^{-1}$ on ice using 20 second bursts of ultrasound (MSE ultrasonicator 6–12 $\mu$ peak height). All sonicates were checked for breakage, the debris spun out at 20,000 rpm (30,000 g) for 1 h and then sterilised by Millipore (0.22 $\mu$) filtration.

### *Colony assays*

For estimation of bone marrow granulocyte-macrophage progenitors (G/M-$CFU_C$), $5 \times 10^4$ marrow cells were plated in 1 ml of Fischer's medium plus 10% horse or FCS supplemented with 0.5% Bacto agar (Difco Ltd) and 20% v/v mouse heart conditioned medium (as a source of granulocyte/macrophage colony stimulating factor, G/M-CSF) in 3 cm diameter plastic petri-dishes. Plates were set up in triplicate and counted after 5 or 7 days incubation at 37° in an atmosphere of 5%

$CO_2$ in air. Colonies containing >50 cells were scored under direct illumination with a colony-counting microscope. For estimation of WEHI-3b clonogenic cells, between 1 and $5 \times 10^4$ cells were cultured as above, omitting the heart conditioned medium.

*Cell recirculation studies—Indium labelling*

The FDCP line A2C2, αThyl negative, Mac-1 and F4.80 positive cell line was used. Cells were washed free of serum and incubated in serum-free RPMI at $10^6$ cells $ml^{-1}$ with 10 μCi $^{111}$Indium Oxime (Radiochemical Centre Amersham, Sp.Act. 370MBq/μgIn) per $10^8$ cells (Thakur *et al.*, 1977). Cells were washed 3 times to remove non-chelated isotope, and $10^7$ cells per mouse injected into normal and irradiated syngeneic recipients via the tail vein. At various time intervals, whole organs (Thymus, Liver, Spleen, Gut, Femur and Lungs) were removed and counted in a γ-scintillation counter. At least 2 mice per time point were used, and $^{111}$Indium labelled normal marrow or lymph-node cells used as controls. Results are expressed as % counts in each organ of the total cumulative counts. The total recovery of label in marrow, gut, lungs, thymus and liver was between 60 and 65% at 24 h.

*Suppression of FDCP and absorption of IL-3 activity by liver cells* in vitro

(a) *Effect of liver cells on FDCP proliferation:* FDCP cells ($10^5$ per well) were co-cultured with various ratios of irradiated liver cells obtained by mechanical dissociation, in Fischer's/FCS medium with 10% WEHI-3b CM. After 24 h, cell proliferation was assayed by [$^3$H]dT. In parallel, FDCP were assayed for clonal growth in agar in the presence of WEHI-3b CM and various ratios of liver cells.

(b) *Absorption of IL-3 activity:* normal liver fragments were treated with collagenase (Type IV, SIGMA, 10 μg $ml^{-1}$ for 1 h at 37°C followed by gentle trypsin digestion (0.025% Trypsin in 0.005% EDTA) for 1 h at 37°C. Remaining clumps were mechanically dissociated, after termination of trypsin treatment by addition of 10% FCS. Approximately $\frac{1}{3}$ liver equivalent of dissociated liver was centrifuged at 100 rpm for 15 min and the cell pellet retained. The supernate was centrifuged at 40,000 rpm (100,000 g) for 1 h. Both cell pellets and supernate were separately incubated with 5 ml 50% WEHI-3b CM at 37°C. Samples were removed at known intervals, centrifuged at 10,000 g for 4 min, filtered and titrated for IL-3 activity against an FDCP line and a factor-independent line, 15al, for non-specific toxicity.

*Co-culture of WEHI-3b cells with normal haemopoietic cells*

Washed WEHI-3b cells ($10^4$ $ml^{-1}$) were co-cultured with $10^4$, $10^5$ or $10^6$ $ml^{-1}$ normal marrow or spleen cells. Samples of the supernates were withdrawn daily, filtered and assayed for IL-3 activity at 10% concentration.

*Half-life of IL-3 activity* in vivo

A preparation of partially-purified factor with very high IL-3 activity was kindly provided by Dr. G. Bazill (Bazill *et al.*, 1983). An amount equivalent to approximately 10 ml WEHI-3b CM (active at 10%) was injected i.v. into a number of normal mice from which serum was collected at known intervals by heart puncture.

*Cell lines*

FDCP-7 and -2 have been previously described (Dexter *et al.*, 1980; Garland & Dexter, 1982*a*). The lines A2C2 and A1.1.1.C2 are Thyl.2 negative FDCP generated from long-term bone marrow culture (LTBMC) at 4 weeks (Garland & Dexter, 1982). All the cell lines are promoted by IL-3 and are refractory to T cell growth factor, GM/CSF and other known cell proliferation factors (Garland & Dexter, 1982*b*). The cell line 15al is a sub-clone of the factor-independent line 416B, also derived from LTBMC (Dexter *et al.*, 1979).

*Fractionation of IL-3 activity on Biogel P30*

Samples (0.2 ml) were fractionated on a 28 × 1.2 cm diameter Biogel P30 (Biorad Labs Ltd) polyacrylamide column equilibrated in PBS (0.85% NaCl; 0.01 M phosphate) and run at 2 ml $hr^{-1}$ at room temperature. Fractions of 0.32 ml were collected and assayed at a final concentration of 25%. The column was calibrated with β lactoglobulin (M.W. 35 Kd) and horse myoglobulin (M.W. 18,800 Kd) (Sigma).

*Proliferative response of WEHI-3b cells to IL-3*

All media was warmed and all operations carried out at 37°C to avoid cold shock. An actively-growing culture of WEHI-3b in Fischer's 10% FCS was obtained by subculture 48 h previously at low cell density ($5 \times 10^4$ $ml^{-1}$). The culture was centrifuged gently (800 rpm, 5 min) resuspended in sufficient Fischer's/10% FCS to yield a density of $10^6$ cells $ml^{-1}$, divided into 2 equal portions and centrifuged as before. One portion was resuspended in fresh Fischer's/10% FCS, the other in the original supernate at 50% v/v, and both returned for re-incubation. At various times, an equal aliquot was removed from each, and assayed for

[$^3$H]dT incorporation (triplicate wells, $10^5$ cells per well, 1 h exact pulse time at 5 $\mu$Ci ml$^{-1}$), cell count, and cell viability by Trypan blue. Results are expressed as the percentage of the values for each culture at the beginning of the re-incubation.

*Animals*

C57Bl/6 and (C57b16 x DBA/2)$F_1$ mice were obtained from the Paterson Laboratories Animal Unit.

### Results

*IL-3 activity in the mice undergoing acute GVHD*

Mice were sampled at various times after GVHD induction. No activity was found in the serum at any stage, and titration of pooled GVHD serum showed no significant inhibition of IL-3 responses at levels below 20% serum (see below). Spleen cells were then cultured with and without PWM, and the supernates tested for IL-3 (Table I). No activity was found when the cells were cultured alone, but at all stages of GVHD, as indicated by spleen index, high concentrations of IL-3 were induced by PWM *in vitro* which titrated down to 5% CM or less. Thus, the cells were still competent to make it. Histology of the spleens at these time-points showed pathologies consistent with moderate to severe GVHD reactions.

**Table I** IL-3 activity in spleen-cell conditioned media from mice undergoing GVH reactions, measured by [$^3$H]dT incorporation

| *Time* | *Spleen index*[a] | *−PWM*[b] *% of control* | *+PWM % of control* |
|---|---|---|---|
| Day 2 | 1.21±0.27 | 3[c] (N.S.) | 148 |
| 4 | 1.52±0.38 | 2 (N.S.) | 137 |
| 6 | 2.1 ±0.21 | 8 (N.S.) | 72 |
| 10 | 1.74±0.63 | 4 (N.S.) | 87 |
| 15 | 1.81±0.23 | 3 (N.S.) | 120 |

[a]Index

$$= \frac{\text{Experimental spleen mass/Experimental mouse mass}}{\text{Control spleen mass/control mouse mass}}.$$

[b]Cells from 3 pooled spleens were cultured 3 days with and without PWM.

[c]Figures are percent of a normal control stimulated with PWM N.S. = not significantly different from control without PWM by $\chi^2$ test. Activity was assayed on the FDCP-2 line, using a dilution of 25% v/v supernates. Absolute levels of [$^3$H]dT incorporations in the positive controls were similar to those described elsewhere (Garland & Dexter, 1982*a*) and in the range 18–30,000 cpm $10^{-5}$ cells.

*IL-3 activity in WEHI-leukaemic mice*

(a) *Establishment of leukaemia* The cloning efficiency of cultured WEHI-3b cells in agar was ~3%, however, this value varied between 0.3 and 6%. With any given culture, clonogenicity in agar was linear between $10^4$ and $10^5$ cells per plate. Below $10^4$ cells there was progressive loss of colonies; above $10^5$ cells counting was difficult because of high background and the presence of many small cell clusters (10–50 cells). Generally at least $10^5$ WEHI-3b cells per mouse were required for leukaemia induction. Most mice injected with $10^6$ WEHI-3b cells died of their leukaemias within 23 days and had greatly enlarged, smooth spleens. The liver also had numerous surface nodules. The numbers of clonogenic cells in marrow and spleens at various injected doses of cells were determined at different times. Table II shows that at 18 days the numbers of clonogenic cells in leukaemic organs was dose-dependent on injected WEHI-3b cells and further that the cloning efficiency had increased; therefore, experiments were standardised to $10^6$ injected WEHI-3b cells per mouse. With $10^6$ injected WEHI-3b cells most, if not all, the clonogenic cells in 18-day spleens and marrow were WEHI-3b cells, as shown by similar numbers of colonies in the presence and absence of heart-conditioned medium (Table II). The relative absence of normal progenitors responding to G/M CSF in leukaemic organs was explicable on their dilution by leukaemia cells, although they were still present (Table II, $10^5$ injected WEHI-3b cells). Thirty individual agar colonies were picked at random from leukaemic spleens and sub-cultured. All clones grew spontaneously and made IL-3. Three cultures were karyotyped and had karyotypes of WEHI-3b

**Table II** GM-CSF-dependent and -independent colonies[a] from 18-day WEHI-3b leukaemic marrow and spleen

| *Source of cells* | *Colonies/3 x $10^4$ with GM-CSF*[b] | *Colonies at 8 days without GM-CSF* |
|---|---|---|
| Normal marrow | 21±4.7 | 0 |
| WEHI-3b culture | 122±15 | 89±15.5 |
| Marrow, +$10^5$ injected WEHI-3b cells | 7.6±3 | 0 |
| Marrow, +$10^6$ injected WEHI-3b cells | 158±11.4 | 144±59 |
| Spleen, $10^5$ injected WEHI-3b cells | 200±51 | 127±34 |
| Spleen, +$10^6$ injected WEHI-3b cells | 200+ | 200+ |

[a]Cells were plated in semi-solid agar. Counts given for mean colonies per plate ±1s.d.

[b]Heart conditioned medium at 20%.

cells (68–73 acrocentrics and one dicentric chromosome). Thus, leukaemic marrow and particularly spleen, had very high numbers of WEHI-3b cells capable of producing IL-3 *in vitro*; based on the cell counts and cloning efficiencies, such leukaemic spleens were calculated to contain at least 85% WEHI-3b cells at 18 days. In the liver, discrete large nodules occurred; cytologically they consisted of virtually pure populations of cells with WEHI-3b morphology. At 18 days, an average of 95 nodules per liver were counted.

(b) *IL-3 activity in leukaemic serum* Examination of the serum from leukaemic mice showed firstly that no IL-3 activity was present at any stage of the disease (Table III). This could be due to inhibitors present in the serum. However, titration curves showed that neither normal nor leukaemic serum significantly inhibited the responses of FDCP to IL-3 (Figure 1) below 10% leukaemic serum concentration in the presence of optimal IL-3 concentrations. Nor was inhibition seen at sub-optimal levels of IL-3 (2.5%). Thus, failure to detect IL-3 in leukaemic serum was not due to the presence of overt inhibitors.

**Figure 1** IL-3 activity in serum of leukaemic mice. Dilutions of serum were assayed against FDCP-7 cells. Control normal serum without WEHI-3b CM gave values closely similar to leukaemic serum; however, the control levels of [$^3$H]dT incorporation in this experiment were higher than normally found, see **Table III**.

**Table III** IL-3 activity in serum[a] from WEHI-3b leukaemic mice

| *Time after Injection (days)* | *No. leukaemic cells injected per mouse* $10^5$ | $10^6$ | $10^7$ |
|---|---|---|---|
| 7 | 2,885±674[b] | 3,044±510 | 2,669±177 |
| 14 | 2,870±651 | 2,396±82 | 4,238±1,544 |
| 18 | 2,428±277 | 2,076±251 | 2,384±406 |
| Control cells+WEHI CM 10% | | 36,582±2,087 | |
| Control cells−WEHI CM | | 2,616±290 | |

[a]Serum was used at 5% final concentration (see **Figure 1**).

[b][$^3$H]dT incorporation in standard assay, cpm±s.d. using FDC-P7.

**Figure 2** IL-3 activity in serum of mice injected i.v. with semi-purified IL-3. (△——△) serum dilutions in the presence of 2.5% WEHI-3b CM (Control). (□——□) serum dilutions 10 min after injection *in vivo* (no WEHI-3b CM). No activity in injected serum was demonstrable after 1 h. Activity was measured by [$^3$H]dT incorporation by FDCP-7; background incorporation without WEHI-3b CM was <2000cpm (in the presence of control serum).

(c) *Half-life of IL-3* in vivo A lack of detectable serum IL-3 could be explained by rapid serum clearance. To test this, a concentrated, partially purified IL-3 (equivalent to ~10 ml WEHI-3b CM active at 10%) was injected i.v. into a number of mice, and the serum collected at various intervals (Figure 2). This serum was then compared with serum to which 2.5% WEHI-3b CM had been added. Whilst activity was present at 10 min post-injection, none was detected at 1 h. The reduction in activity after 10 min *in vivo* compared to serum *in vitro* extrapolates to a half-life of <30 min. Therefore, the absence of detectable IL-3 in leukaemic serum could be partially accounted for by its very short half-life *in vivo*.

(d) *Detection of intracellular IL-3 in leukaemic organs* With the above result, it was decided to determine if IL-3 was in fact made *in vivo* at all. WEHI-3b produces IL-3 constituitively *in vitro* and its presence intracellularly was readily demonstrable in centrifuged sonicates of washed WEHI-3b cells (Table IV) However, when similar numbers of 18-day leukaemic spleen cells were sonicated, no

**Table IV** Intracellular IL-3 in sonicates of WEHI-3b cells grown *in vitro* and *in vivo*: comparison with IL-3 in WEHI-3b conditioned medium

| Cell population | | Cell no. equivalent/ml of sonicate | % sonicate in test[a] v/v | % of control counts[b] in sonicate: without WEHI-3b CM | with 10% WEHI-3b CM |
|---|---|---|---|---|---|
| WEHI, *in vitro* | | $2.3 \times 10^7$ | 50 | 78 | ND |
| | | | 12.5 | 49 | ND |
| Leukaemic marrow | 1 | $0.8 \times 10^7$ | 50 | 2.6 | 105 |
| (18 days) | 2 | $10^7$ | 50 | 1 | 73 |
| Leukaemic spleen | 1 | $4.3 \times 10^7$ | 50 | 1 | 72 |
| (18 days) | 2 | $4 \times 10^7$ | 50 | 1 | 93 |
| Leukaemic nodules in liver | | $2.5 \times 10^7$ | 6 | 5.3 | 4.6[c] |
| (18 days) | | | 3 | 30 | 44 |

[a]Sonicates assayed on FDCP.
[b]Control counts = cell grown in 3% WEHI-3b CM (1% above optimum concentration).
[c]Leukaemic nodules were assayed on FDCP A1.1.1.C2. See **Table VII** also.

activity was detectable in the sonicates either by viability estimations on, or [$^3$H]dT incorporation by, FDCP indicator cells. Table IV also shows that at a 5-fold excess of centrifuged sonicates over WEHI-3b CM none of the sonicates from leukaemic spleen and marrow contained substances inhibiting the assay (see below). Further, in the absence of WEHI-3b CM, residual [$^3$H]dT incorporation by FDCP was maintained showing that the sonicates were not directly toxic to the cells. That cells from leukaemic organs were still able to elaborate the factor was shown by incubating small numbers of leukaemic spleen cells ($10^4$ ml$^{-1}$) *in vitro,* when IL-3 was again readily detectable in the culture supernates (Table V). Further, normal spleen and marrow cells did not inhibit the production of, or markedly absorb, IL-3 *in vitro* as shown in Table VI; very high concentrations of normal spleen and marrow cells were co-cultured with WEHI-3b cells without producing a significant drop in IL-3 titre, particularly during the early synthetic phase when inhibition or absorption would have greatest effect. It was therefore important to determine if this apparent lack of IL-3 synthesis *in vivo* was common to all leukaemic cells regardless of anatomical site. Sonicates of leukaemic nodules from the livers at 18 days contained significant IL-3 activity but it was masked at high sonicate concentrations by inhibitors (Table IV). The level of activity in sonicates was approximately 5-fold less than that in a similar quantity of WEHI-3b cells grown *in vitro.* Thus, liver nodule sonicates contrasted with those from leukaemic marrow and spleen in the presence of both IL-3 and inhibitors. Having demonstrated production *in vivo* of IL-3 within compact WEHI-3b colonies, mice were taken to 21 days and spleen and marrow sonicates examined for IL-3 (Table VII). In contrast to the previous results, IL-3 activity was now detectable in spleen sonicates, but the levels were low (more than 10-fold lower than in *in vitro* sonicates) and there was indication of the presence of inhibitors. To determine whether the inhibition was specific to IL-3 dependent cells, the same sonicates were titrated against the factor-independent haemopoietic cell line 416B. The results showed that the inhibition was not specific as the factor-independent line was also inhibited (Table VII). Visual examination of the wells showed that the [$^3$H]dT incorporation reflected cell viability and proliferation and not competition for isotope by small molecular weight nucleotides released during sonication.

**Table V** IL-3 activity in conditioned medium from 18-day leukaemic spleen or marrow, cultured 3 days[a] *in vitro*

| % concentration CM | Cultured spleen[b] CM | Cultured marrow CM |
|---|---|---|
| 20 | 36,530 ± 4,314 | 42,978 ± 1,822 |
| 5 | 23,462 ± 5,136 | 22,073 ± 1,293 |
| 1 | 17,579 ± 2,139 | 11,465 ± 1,426 |
| Control medium with 10% WEHI-3b CM | | 36,582 ± 2,087 |
| Control medium without WEHI-3b CM | | 2,616 ± 290 |

[a]Without mitogen. No activity was present in normal spleen or marrow cultures without mitogen.
[b]Counts given for mean cpm ± 1s.d.

**Table VI** Normal spleen and marrow cells do not suppress *in vitro* production of WEHI-3b cell derived IL-3

| *No. spleen marrow cells* $ml^{-1}$ | *No. co-cultured WEHI cells* $ml^{-1}$ | *Day 1 S–N* | | *Day 2 S–N* | |
|---|---|---|---|---|---|
| | | *Marrow* | *Spleen* | *Marrow* | *Spleen* |
| $10^4$ | $10^4$ | 2,833±218 | 3,309±149 | 9,187±620 | 6,011±737 |
| $10^5$ | $10^4$ | 3,223±96 | 2,975±195 | 7,709±1,213 | 7,771±588 |
| $10^6$ | $10^4$ | 3,078±67 | 4,153±263 | 9,332±367 | 1,1051±1,638 |
| 0 (control) | $10^4$ | 3,309±395 | | 579±739 | |
| 0 (Control without IL-3) | 0 | 1,377±334 | | — | |
| 10% WEHI-3b CM control | | 18,862±1,971 | | — | |

Day 3 assay samples showed levels of IL-3 activity equal to 10% standard WEHI-3b CM in cell supernates (S–N).

**Table VII** Intracellular IL-3 in sonicates of 21-day leukaemic nodules[a] and spleens[b]

| *% sonicates* | *Liver nodule* | *Liver nodule + 3% WEHI-3b CM* | *Spleen* | *Spleen + 5% WEHI-3b CM* | *416B+ liver sonicates* | *416B+ spleen sonicates* |
|---|---|---|---|---|---|---|
| 12.5 | 1,201±186 | 1,274±100 | 3,586±686 | 7,898±640 | 570±367 | 34,366±2,646 |
| 6 | 2,716±181 | 2,358±286 | 8,409±1,028 | 16,678±842 | 481±16 | 52,312±4,288 |
| 3 | 14,976±1,049 | 22,157±466 | 6,856±354 | 19,094±1,575 | 2,781±342 | 65,187±2,775 |
| 1.5 | 10,286±2,600 | 39,634±1,138 | 4,241±511 | 21,728±954 | 30,760±8,723 | 61,432±3,208 |
| S-480-3 control | 50,435±6,386 | — | 28,114±888 | — | — | — |
| S-480-3 control no. WEHI-3b CM | 1,544±41 | — | 910±34 | — | — | — |
| 416B control | — | — | — | — | 74,698±139 | — |

[a]Sonicates were assayed on FDCP line A1.1.1.C2. Individual leukaemic nodules were dissected out from the liver; a total of 160 mg wet weight, equivalent to $5 \times 10^7$ cells, were used in 2 ml.
[b]Figures are for one 700 mg wet weight spleen, equivalent to $\sim 5 \times 10^8$ cells, in 2 ml.
[c]Figures are given for cpm ±s.d. on duplicate wells.

### *Molecular weight fractionation of* in vivo *IL-3*

It was possible that *in vivo* IL-3 activity was associated with a molecular species different from *in vitro* IL-3. Liver nodule sonicates were therefore fractionated on Biogel P30, the elution pattern being compared with that of WEHI-3b CM and a preparation IL-3. IL-3 eluted with an apparent average mol. wt of 28 Kd, (Figure 3) but recovery was low; ~80% was irreversibly bound (2 experiments). However, a similar mol. wt average for IL-3 has recently been given (Ihle *et al.*, 1982). In common with previous experience (Bazill *et al.*, 1983) WEHI-3b CM eluted over a broader slightly higher mol. wt range with an average of 32 Kd (mean peak height). Liver sonicate activity eluted almost identically to IL-3 (with an enhanced shoulder at 32 Kd). The elution characteristics of the *in vivo* activity therefore were closely similar to IL-3 and WEHI-3b CM.

### *Studies on the circulation of FDCP*

We wished to know if factor-dependent cells represented a normal cell population. Experiments were therefore performed to determine if cells from a dependent line were viable after injection into (a) normal and (b) lethally (8.5 Gy) irradiated recipients, in (b) recognising them by their ability to subsequently grow in WEHI-3b CM supplemented agar or liquid culture. When up to $10^7$ FDCP-1 cells were injected per irradiated mouse, none could be recovered from marrow or spleen even after 30 min post-injection and up to 24 h later. It was possible that the FDCP were rapidly killed *in vivo*, and therefore studies were undertaken in which an FDCP line (A2C2) with a "half-life" (by [$^3$H]dT incorporation) of about 15 h in the absence of IL-3 was labelled with non-reutilisable $^{111}$Indium and the cell's circulation in normal and lethally irradiated mice followed. The results are given in

**Figure 3** Fractionation of IL-3 activities on Biogel P30 polyacrylamide column. Samples were eluted with PBS. The void volume as estimated by blue dextran dye was 4.6 ml, at position 14/15. Markers are given for $\beta$-lactoglobulin and horse myoglobin (35 and 18,800 Kd). Recoveries were >80% for WEHI-3b CM and liver sonicate; recovery of IL-3 was ~20%.

Figures 4 & 5. Firstly, normal marrow cells were used as controls in normal mice (Figure 4); these showed a rapid traffic of label between spleen and lungs over the first 8 h. Only small numbers of cells (<2%) were found in the marrow, and virtually none in thymus. Most cells apparently lodged in the liver eventually (at ~12 h). The circulation of FDCP, was, however, quite different (Figure 5). There was progressive re-circulation from the lungs to both spleen and liver; this continued over a period of nearly 20 h, and was not complete until after this time. Approximately the same number of FDCP lodged in the marrow, gut and thymus as normal marrow cells. Similar results were recorded in lethally-irradiated recipients (data not shown). Thus, although no FDCP were recovered in the previous experiments the cells were still recirculating *in vivo.*

### *Suppression of FDCP by liver cells* in vitro

Since the above data showed that FDCP were arrested in the liver, we determined whether liver cells could suppress FDCP or metabolise IL-3 *in vitro.* In the first experiments, FDCP were mixed with various ratios of normal and irradiated liver cells in an agar colony assay. At ratios of 1

**Figure 4** Recirculation of $^{111}$Indium-labelled normal marrow cells in normal mice cells. Note reciprocal exchange between spleen and lungs at 2–4 hours post-injection. Points given for mean counts ±s.d.

**Figure 5** Recirculation of $^{111}$Indium-labelled FDCP (line A2C2) in normal mice. Compare with **Figure 2.** Note progressive accumulation of counts in liver and spleen and reciprocal loss from lungs.

FDCP: 2000 liver cells, colony formation was completely inhibited, (Table VIII) although at lower ratios (1:400) colonies were observed. In WEHI-3b CM supplemented liquid culture, FDCP apparently survived for at least 2 days as judged by phase microscopy when co-cultured with ratios of 1:1000 and 1:5000 liver cells, but despite repeated subculturing, none were subsequently recovered. These results could be due to absorption of the IL-3 by liver cells. Initial results using unseparated liver suspensions to absorb WEHI-3b CM suggested that IL-3 activity was very rapidly lost (none at 4 h incubation). Experiments were therefore performed using liver suspensions separated by centrifugation into a cell-free supernate and two sizes of pellet. These were used to absorb excess and sub-optimal amounts of IL-3, the supernates being titrated against both an FDCP line and a factor-independent line, 15al. Titrations of the absorbed supernates showed that by both [$^3$H]dT assay and viability estimation, considerable inhibitory activity on both the FDCP line and 15al factor-independent line was present in all samples after 2 h incubation particularly in the 100,000 g supernate. However, the IL-3 activity was unchanged and re-appeared at higher dilutions of supernates (Table IX). As the viability of the initial liver cells suspensions was low (15–20%), these results show that *in vitro* neither liver cells nor their released contents contain degradative enzymes or inhibitors specific to IL-3 and the inhibition of FDCP cells *in vitro* previously found with liver cells was probably due to non-specific toxicity.

**Table VIII** Suppression of FDCP colonies in agar by normal liver cells

| *Ratios of FDCP to liver cells*[a] | *No. colonies at 7 days* |
|---|---|
| $1:25\times10^5$ | 0 |
| $1:6.4\times10^4$ | 0 |
| 1:800 | $106\pm9$ |
| 1:0 Control | >500 |

[a] $5\times10^4$ FDCP2 cells plated.

### *Response of WEHI-3b cells to IL-3*

If WEHI-3b cells respond to extracellular IL-3, we reasoned that actively-growing cells replaced in fresh medium may suffer an extended early lag period whilst sufficient IL-3 accumulates to restimulate growth. We therefore compared cells replaced in fresh medium with those in 50% of their own conditioned medium, Figure 6. However, this showed that there was no difference in the early proliferative responses of cells with or without added IL-3; the two curves were parallel and almost superimposed. Over the first few hours of re-culture, proliferation in both cultures decreased

**Table IX** Incubation of WEHI-3b CM with liver cells; effect on IL-3 activity after 2 hours[a]

| *Conditioned medium* | *Cell line assayed* | *% liver-conditioned medium[b] in assay; cpm ± s.d.* 25 | 12 | 6 |
|---|---|---|---|---|
| Cell pellet | 15al | 10,640 ± 1,275 | 24,159 ± 154 | 40,113 ± 1,989 |
| | A1.1.1.C2 | 13,007 ± 1,751 | 15,460 ± 2,265 | 17,018 ± 1,266 |
| 100,00 g pellet | 15al | 11,270 ± 3,165 | 29,691 ± 2,519 | 47,605 ± 5,776 |
| | A1.1.1.C2 | 12,496 ± 1,104 | 17,840 ± 1,466 | 18,793 ± 1,217 |
| 100,00 g S–N | 15al | 779 ± 280 | 1,155 ± 436 | 5,980 ± 132 |
| | A1.1.1.C2 | 976 ± 116 | 13,524 ± 373 | 20,874 ± 653 |
| Control, no liver cells | 15al | 45,290 ± 1,044 | 58,169 ± 457 | 6,669 ± 6,107 |
| | A1.1.1.C2 | 19,155 ± 369 | 20,617 ± 50 | 18,781 ± 613 |

[a]Similar activities recovered after 18 h incubation.
[b]Original IL-3 in the conditioned media; concentration was 12, 6 and 3% respectively in the assay.

**Figure 6** Proliferation of WEHI-3b cells after transfer to fresh medium with and without IL-3. Initial viabilities were 99%, and T = 0 incorporation 18,000 cpm.

significantly; after 24 h in fresh medium proliferation had increased, and logarithmic growth started; but cells replaced in the conditioned medium were still depressed. Viability in both cultures paralleled the thymidine uptake, but the effect was much less; the maximum drop was not more than 25% at 2 h (data not shown).

### Discussion

The purpose of these studies was to determine the presence of IL-3 *in vivo* and the fate of IL-3 dependent cell lines. Parallels exist for other haemopoietic growth factors, for example, G/M CSF and TCGF. CSF is elaborated *in vivo*, as it is readily obtained from sources such as urine and serum (Moore *et al.*, 1974; Das *et al.*, 1981; Burgess & Metcalf, 1980), although injected CSF has a short life (Shadduck *et al.*, 1980). Additionally, radiolabelled CSF has some affinity for the haemopoietic tissue on which it acts (Shadduck *et al.*, 1980). In terms of target cells, CSF promotes both proliferation and differentiation of myeloid progenitors (Burgess & Metcalf, 1980) such that functional cells are produced *in vitro* (Gorczynski *et al.*, 1980). Likewise, activated T-cells have receptors for IL-2, which appears to be a proliferation-mediator without differentiating ability (Raulet & Bevan, 1982). Although much is now known about cellular mechanisms related to IL-2 promoted proliferation *in vitro* (Maizel *et al.*, 1981; Sekaly *et al.*, 1982), its production *in vivo* is still uncertain; this may be related again to its short half-life *in vivo* and the presence of inhibitors in serum (Wagner *et al.*, 1980; Bindon *et al.*, 1982). However, injection of IL-2 into immunodeficient animals (nu/nu mice) appears to result in establishment of T-cell like responses suggesting that it may act physiologically (Wagner *et al.*, 1980); a similar conclusion has been made for the myeloid differentiation factor described by Lotem & Sachs (1981). Our experiments are different in that we have used a constituitive (*in vivo*) lymphokine-producing leukaemic cell line. We found intracellular lymphokine in early leukaemic liver nodules, and have shown that it is identical or very closely similar to IL-3 produced *in vitro*. Although we did not find it initially in corresponding marrow or spleen lysates, nor in serum, its presence intracellularly in spleens from terminal leukaemic mice suggests that the problem in earlier lysates is logistical; the amount *in vivo* is considerably less than in a corresponding number of cells grown *in vitro*. Further, the intracellular presence of IL-3 is correlated with the presence of potent inhibitors which mask its activity *in vitro* and suppress other cells. This may be relevant to the requirement for a minimal number of WEHI-3b cells to induce leukaemia, and the long latent period before the leukaemia becomes overt; *in vitro*, the mass-generation time of WEHI-3b cells is about

18 h, and at this proliferation rate a 15-day leukaemic mouse would greatly exceed its own body-weight in leukaemic cell load.

Our experiments thus show conclusively that IL-3 is not an *in vitro* artifact; and suggest that its production may be related to the leukaemic state. Thus, the factor (or a group of very similar ones) promotes many cell lineages *in vitro* (Garland & Dexter, 1982*b*; Bazill *et al.*, 1983; Garland & Crompton, 1983; Iscove *et al.*, 1982) and "immortalises" cell lines with characteristics of the WEHI-3b leukaemic line (Dexter *et al.*, 1980). "Misplaced" elaboration of IL-3 internally could therefore be a universal route to leukaemia induction within a range of haemopoietic cells. In WEHI-3b cells, such a mechanism does not appear to involve surface receptors, as if actively-growing WEHI-3b cells are replated in WEHI-3b CM, there is no enhancement of proliferation compared to controls without WEHI-3b CM; both cultures suffer equal falls in [$^3$H]dT incorporation before growth is resumed. The reason for the initial fall is not clear; we have consistently observed a similar effect in FDCP replated in fresh medium with IL-3, and it appears to be related to the physical manipulation of the cells. The apparent continuing depression in the culture with conditioned medium does not seem to be entirely explicable on medium quality (50% conditioned medium) as firstly, the cells were actively growing in it at 100% and secondly, the dilution by fresh medium should have allowed at least one doubling over 18 to 20 h (maximum culture density of WEHI-3b cells is $\sim 2 \times 10^6$ cells ml$^{-1}$).

In contrast to leukaemic mice, *in vitro* culture of lymphocytes activated *in vivo* by GVH reactions is not associated with IL-3 production. This is somewhat surprising as in early GVH reactions a significant component of the proliferation is host cell (Ford *et al.*, 1981), presumably mediated by soluble factors *in vivo* (English, 1982). With regard to the inability to recover FDCP after *in vivo* injection, the recirculation and co-culture experiments suggest that one major problem is non-specific cell toxicity generated by normal cells e.g. liver, *in vitro*. Thus, the number of normal cells needed to be harvested to ensure adequate representation of FDCP cells is more than sufficient to inhibit their growth; in addition IL-3 is rapidly cleared *in vivo* and FDCP are sensitive to factor withdrawal (Garland & Dexter, 1982*b*). The most likely explanation is therefore that FDCP neither proliferate nor survive long enough to recover. However, there is a report of similar WEHI-3b CM dependent lines maturing *in vivo* in diffusion chambers (Greenberger *et al.*, 1980). It therefore seems paradoxical that FDCP lines can be generated at all; all the lines have been derived from long-term marrow cultures, which are defective in T-cells (Phillips, 1980; Schrader *et al.*, 1979). Marrow cultures do not elaborate detectable levels of IL-3 but can be induced to do so if T-cells are supplied (J. Garland, in preparation). If the lines are absolutely dependent on IL-3, then either IL-3 is elaborated at micro-levels in the cultures, in a manner similar to G/M CSF (Shadduck *et al.*, 1980), or FDCP represent lines that do not normally arise but are "diverted" to IL-3 dependency by culture selection. This seems unlikely as firstly, IL-3 which is very closely similar if not identical to HCGF, promotes other recognisable cell lineages *in vitro* (Bazill *et al.*, 1982; Garland & Crompton, 1982), and secondly, other cell lines dependent on added factors can function *in vivo* as in the case of cloned NK lines (Warner & Dennert, 1982). Apparently, GVH mice do have increased numbers of IL-3 promoted progenitors (J. Schrader, personal communication) and studies to determine the fate of IL-3 dependent lines in WEHI-3b leukaemic mice are in progress.

Finally, although FDCP do not seem to be related to T-cells themselves, they respond to a T-cell associated factor and it is tempting to speculate that one set of T-cell functions may be to assist in the regulation of haemopoiesis, unrelated to their undoubted function in immunity. Support for the notion can be found in numerous examples of T-cell help for proliferation of $CFU_S$ and indeed the establishment of long-term haemopoietic cultures (Sharp *et al.*, 1981; Dexter *et al.*, 1977).

Our conclusions are that IL-3 is not an *in vitro* artifact and that it may be related to the leukaemic potential of WEHI-3b cells. Problems still remain with identifying the factor as a physiological haemopoietic regulator, but the construction of genetic probes and antibodies to it offers a fruitful direction to pursue.

This work was supported by the Cancer Research Campaign. We thank Miss Stella Crompton for excellent technical assistances, and Mrs A. Spreadborough for assistance in performing the karyotypes.

**References**

BAZILL, G., HAYNES, M., GARLAND, J.M. & DEXTER, T.M. (1983). Characterisation and partial purification of a hemopoietic factor in WEHI-3b cell conditioned medium. *Biochem. J.*, **210**, 747.

BINDON, C., CZERNIECKI, M., RUELL, P. & 4 others. (1982). Clearance rates and systemic effects of intravenously administered interleukin 2 (IL-2) containing preparations in human subjects. *Br. J. Cancer*, **47**, 123.

BURGESS, P.W. & METCALF, D. (1980). The nature and action of granulocyte/macrophage colony stimulating factor. *Blood*, **56**, 947.

DAS, S.F., STANLEY, E.R., GUILBERT, L.J. & FORMAN, L.W. (1981). Human colony-stimulating factor (CSF-1) radioimmunoassay: Resolution of three sub-classes of human colony-stimulating factors. *Blood*, **58**, 630.

DEXTER, T.M., ALLEN, T.D. & LAJTHA, L.G. (1977). Conditions controlling the proliferation of hemopoietic stem cells *in vitro*. *J. Cell Physiol.*, **91**, 335.

DEXTER, T.M., ALLEN, T.D., SCOTT, D. & TEICH, N. (1979). Isolation and characterisation of a bipotential haematopoietic cell line. *Nature*, **277**, 471.

DEXTER, T.M., GARLAND, J.M., SCOTT, D., SCOLNICK, E. & METCALF, D. (1980). Growth of factor-dependent hemopoietic precursor cell lines. *J. Exp. Med.*, **152**, 1036.

ENGLISH, L.S. (1982). Immunoregulatory actors produced by activated lymph-nodes *in vivo*. In: *In vivo Immunology, Adv. Exp. Biol. Med.*, **149**, 601.

FORD, W.L., ROLSTAD, R., FOSSUM, S., HUNT, S.V., SMITH, M.E. & SPARSHOTT, S.M. (1981). The stimulus to host cell proliferation in graft versus host reactions. *Scand. J. Immunol.*, **14**, 705.

GARLAND, J.M. & CROMPTON, S. (1983). Preparations containing Interleukin 3 (IL-3) promote proliferation of multi-potential stem cells (CFU-S) in the mouse. *Exp. Haematol.*, (in press).

GARLAND, J.M. & DEXTER, T.M. (1982*a*). 20α hydroxysteroid dehydrogenase expression in hemopoietic cell cultures and its relationship to interleukin 3. *Eur. J. Immunol.*, **12**, 998.

GARLAND, J.M. & DEXTER, T.M. (1982*b*). Lymphoid antigens on non-lymphoid factor-dependent hemopoietic cell lines. In: *In vivo Immunology, Adv. Exp. Biol. Med.*, **149**, 127.

GARLAND, J.M., DEXTER, T.M., LANOTTE, M., HOWARTH, C. & ALDRIDGE, A. (1983). Relationship of WEHI growth factor (WGF), Interleukin 3 (IL-3) and 20α steroid dehydrogenase (20αSDH) to hemopoietic progenitor cells. In: *Proceedings, 3rd Int. Conference Lymphokines*, (Eds. Oppenheim & Cohen) (In Press).

GARLAND, J.M. & DEXTER, T.M. (1983). Relationship of Haemopoietic Growth Factor to lymphocytes and Interleukin-3; a short review. *Lymphokine Res.*, **2**, 13.

GORCZYNSKI, R.M., BENZING, R., MACRAE, S. & PRICE, G. (1980). *In vivo* generation from murine bone marrow cells of accessory cells with discrete antigen presentation capacity and ability to release lymphostimulatory molecules. *Immunopharmacology*, **2**, 327.

GREENBERGER, J.S. (1980). Self-renewal of factor-dependent hemopoietic progenitor cell lines derived from long-term bone marrow cultures demonstrate significant mouse strain genotypic variation. *J. Supramol. Struct.*, **13**, 501.

GREENBERGER, J.S., ECKNER, R.J., OSTERTAG, W. & 6 others. (1980). Release of spleen focus-forming virus (SFFV) from differentiation inducible promyelocytic leukaemic cell lines transformed *in vitro* by Friend leukaemia virus. *Virology*, **105**, 425.

HAPEL, A., LEE, J.C., FARRAR, W.L. & IHLE, J. (1981). Establishment of continuous cultures of Thy 1.2 positive, Lyt 1 positive 2 negative T-cells with purified interleukin 3. **Cell**, **25**, 179.

IHLE, J., KELLER, J., HENDERSON, L., KLEIN, F. & PALAZYNSKI, E. (1982). Procedures for the purification of Interleukin 3 to homogeneity. *J. Immunol.*, **129**, 2431.

IHLE, J., REBAR, L., KELLER, J., LEE, J.C. & HAPEL, A. (1982). Interleukin-3: Possible roles in the regulation of lymphocyte differentiation and growth. *Immunol. Rev.*, **63**, 532.

ISCOVE, N.,ROITSCH, C.A., WILLIAMS, N. & GUILBERT, L.J. (1982). Molecules stimulating early red cell, granulocyte, macrophage and megakaryocyte precursors in culture; similarities in size, hydrophobicity and charge. *J. Cell Physiol.*, (Suppl. 1) 65.

LARSSON, E.K., ISCOVE, N. & COUTINHO, A. (1980). Two distinct factors are required for the induction of T-cell growth. *Nature*, **283**, 664.

LOTEM, J. & SACHS, L. (1981). *In vivo* inhibition of the development of myeloid leukaemia by injection of macrophage- and granulocyte-inducing protein. *Int. J. Cancer*, **28**, 375.

MAIZEL, A.L., SHASHKANT, P., MEHTA, A. & 4 others. (1981). Human T lymphocyte/monocyte interaction in response to lectin; kinetics of entry into S-phase. *J. Immunol.*, **127**, 1058.

METCALF, D. (1981). Control of Haemopoietic Cell proliferation and differentiation. In: *Control of Cell Division and Development, Part A*, p. 473, Alan Liss Inc.: New York.

MOORE, M.A.S., SPITZER, G., METCALF, D. & PENINGTON, D.G. (1974). Monocyte production of colony stimulating factor in familial cyclic neutropenia. *Br. J. Haematol.*, **27**, 47.

PHILLIPS, R.A. (1980). Enhanced lymphoid and decreased myeloid reconstituting ability of stem cells from long term cultured mouse bone marrow. *J. Immunol.*, **124**, 597.

RAULET, D. & BEVAN, M.J. (1982). A differentiation factor required for the expression of cytotoxic T-cell function. *Nature*, **296**, 754.

SCHRADER, J., GOLDSCNEIDER, I., BOLLUM, F.J. & SCHRADER, S. (1979). *In vitro* studies of lymphocyte differentiation. II. Generation of TdT positive cells in long-term cultures of bone marrow. *J. Immunol.*, **122**, 2337.

SCHRADER, J., LEWIS, S.J., CLARK-LEWIS, I. & CULVENOR, J.A. (1981). The persisting cell: Histamine content regulation by a cell-derived factor, origin from bone-marrow precursors and relationship to mast cells. *Proc. Natl Acad. Sci.*, **78**, 323.

SEKALY, R.P., MACDONALD, H.R., ZAECH, P. & NABHOLZ, M. (1982). Cell cycle regulation of cloned cytotoxic T cells by T-cell growth factor; analysis by flow microfluorimetry. *J. Immunol.*, **129**, 1407.

SHADDUCK, R.K., PIGOLI, G., WAHEED, A. & ROEGEL, F. (1980). The role of colony stimulating factor in granulopoiesis. *J. Supramol. Struct.*, **14**, 423.

SHARP, J.G., ANDERSON, R.W., CROUSE, D. & CULLEN, G.M. (1981). Effects of thymus and thymic factors on the number and proliferative status of murine haemopoietic stem cells. In: *Experimental Haematology Today*, p. 127, (Eds. Baum *et al.*) Karger Ltd: New York.

THAKUR, M., SEGAL, A.W., LOUIS, L., WELCH, M.J., HOPKINS, J. & PETERS, T.J. (1977). Indium 111 labelled cellular blood components: mechanism of labelling and intracellular location in human neutrophils. *J. Nucl. Med.*, **18,** 1022.

WAGNER, H., HARDT, C., HEEG, K., ROLLINGHOFF, M. & PFITZENMAIER, P.F. (1980). T-cell development helper factor allows *in vivo* induction of cytotoxic T cells in nu/nu mice. *Nature,* **284,** 278.

WARNER, J. & DENNERT, G. (1982). Effects of a cloned cell line with Natural killer activity on bone marrow transplants, tumour development and metastasis *in vivo. Nature,* **300,** 31.

WARNER, N., MOORE, M.A.S. & METCALF, D. (1969). A transplantable myelomonocytic leukaemia in Balb/c mice: Cytology, karyotype and neuraminidase content. *J. Natl Canc. Inst.*, **43,** 963.

YUNG, Y.-P. & MOORE, M.A.S. (1982). Long-term *in vitro* culture of murine mast cells. III. Discrimination of mast-cell growth factor and granulocyte-stimulating factor. *J. Immunol.*, **129,** 1256.